# Surgery of the Paralyses of Children

**Published:** 1903-02-28

**Authors:** 


### SURGERY OF THE PARALYSES OF CHILDREN.

At a meeting of the Huddersfield Medical Society, Mr Robert Jones[1] called attention to the surgical aspects of the paralyses of children, and lamented the neglect which is bestowed on these cases by surgeons generally. He points out that by skill and perseverance a great deal of good may be achieved in some of these cases, but that the surgeon must be thoroughly acquainted with the theory of his subject, and must himself direct the mechanical details which are the accompaniments of all operative aid. He deals first with some aspects of acute infantile paralysis—the most prolific source of deformity next to rickets, the deformity never disappearing spontaneously. Paralytic deformity with shortened muscle or diminished joint movement will not occur under proper treatment. Joint malposition due to muscle shortening arises from insufficiently opposed muscular action or from faulty posture due to the effects of gravity combined with other factors, such as the shape of the articular facets, and he points out that sometimes the shortened muscles are found to be the paralysed ones. In the limb not completely paralysed contractions of the more powerful groups of muscles act very harmfully on their weaker opponents, and it is the recognition of this fact which constitutes the keynote to both mechanical and operative treatment. In the past, Mr. Jones thinks, the pathology of the affection has dominated too much over the clinical aspects. The destruction of the motor nerve cells is not so extensive and complete as it is often assumed to be, and a muscle apparently completely paralysed may regain its power if the evil effect of the opposing muscles is removed for a time. Muscles powerless from desuetude must not be mistaken for muscles paralysed from cell destruction. The method of distinguishing is to move the affected joint into such a position as will relax as far as possible the healthy muscles, and if, in this position, the pseudo-paralysed muscles can cause any movement whatever the case is not hopeless and systematic treatment is indicated. The treatment is to stretch the healthy muscles and relax the weakened ones over a long period of time. No attempt should be made to treat a weakened set of muscles without first rescuing them from being overstretched. After discussing tendon transplantation and arthrodesis Mr. Jones passes on to cerebral paralysis of the spastic type and its treatment. He argues that a large proportion of children suffering from severe spastic paralysis may be transformed into useful members of the community, improved both in body and mind, by surgical efforts, enabled to walk with comparatively little deformity, and generally needing but the aid to be derived from one or two sticks. Active treatment may be required for two years, and it would therefore be unwise to admit a case into hospital for two months and then to send it to a miserable home where neglect would be sure to follow. Treatment of all cases of spastic paralysis is divided into operative and post-operative. With regard to prognosis, if the paralysis is complete and the little patient is never known to relax his spasm, treatment is futile. If he only moves the fingers of the affected hand in conjunction with the fingers of the opposite side the results will probably be discouraging. If any increase of voluntary power is shown the success of treatment is assured. Similarly, when any degree of voluntary relaxation of spasm exists apart from an associated movement on the opposite side, treatment is emphatically indicated. It may be taken as an axiom that prolonged fixation of spastic muscles in a position opposed to their contraction lessens the severity of their spasm. This forms the basis of splint treatment. Operative measures are tenotomy and tendon transplantation. Empiricism has taught that tenotomy of any single tendon controls or lessens not only the spasm affecting its own muscle, but also the spasm affecting the associated muscles. Free tenotomy and fixation in positions opposite to those which the spasmodic contractions cause are the main indications in the treatment of spastic paraplegia. In conclusion, Mr. Jones is convinced that infantile paralysis offers an immense field for surgical enterprise.

[1] Lancet, Feb. 14.

